# Value of Medicine

**Published:** 1868-09

**Authors:** 


### Value of Medicine.

An Inquiry (from a philosophical point of view) concerning its true value in the Treatment of Disease. [Extract from an address by Mr. W. Wilmott to the Chemist's Assistants' Association, May 14, 1868.]

"If we say that a large majority of the medical profession believe in the efficacy, to a greater or less extent, of the drugs they prescribe, we shall not be far wrong. Such a belief has always existed; but it is clear that it presented a very different aspect formerly to that which it presents now. The history of medicine, in fact, reveals to us a strange complication of credulity and superstition. One feature connected with it is singularly noticeable. From the earliest ages down to the present time, there has been an intense desire, with a view either to wealth or fame, to discover one remedy or one law, which shall prove of universal application. Sometimes a fancied discovery of this kind is called a "doctrine," such as the "Doctrine of Signatures," and the "Doctrine of *Similia Similibus Curantur*." Sometimes it is called a "method," such as the "Expectant Method," much in favor, as we have seen, at the present time. Sometimes it is called a "treatment," such as the "Antiseptic Treatment," the "Chrono-thermal Treatment," and the "Eclectic Treatment;" and sometimes it is called a "cure," such as the "Water Cure," the "Movement Cure," and the "Great Sulphur Cure." All these, or the majority of them (true, perhaps, to a limited extent, and, because true, so far successful,) have failed, and always will fail, when indiscriminately or universally applied. No discovery of this kind can or will ever be made, simply because there is no such remedy or law to discover. Disease, like every other phenomenon, is subject to an infinity of laws—if, indeed, they are laws at all, which must be met according to the form and direction which, in each case, they may have assumed. This is the reason why what is called "allopathy," or orthodox medicine, includes within its wide range all the philosophy which, so far as we have yet gone, it is possible to bring into active operation. We shall presently see how it comes to pass that one remedy is so largely applicable, and one mode of treatment so uniformly safe, in a given number of cases.

We need not, I think, go beyond our own shores in search of skill and ability in dealing with disease. I am not aware that in France, America, or elsewhere, the treatment adopted is more successful than that approved of in this country. The system, therefore, as, in a manner, indicated in our national pharmacopœia, is the one to which we look with confidence as the best that can be devised in the present state of our therapeutical knowledge. What, then, are the prominent features of this system in the hands of the orthodox practitioner? In what light is medicine regarded by those of our physicians who retain a welcome and an honest faith in the work of their profession?

In seeking an answer to these questions, with a view to the present inquiry, I adopted a plan which, I have no doubt, you will regard as satisfactory for the purpose intended. From a large number of prescriptions actually dispensed in the city of London, I selected one thousand. These were written by different medical men for different diseases, and different symptoms of disease. They were also written at different seasons of the year, (Spring, Summer, Autumn and Winter,) during a period extending over the past ten years. I did not select them on account of any specialty they possessed, but took them as they were copied, in writing, in the book kept, as usual, for that purpose. As, in this work, my eye passed over many thousands of prescriptions, I was enabled to satisfy myself that those I had selected represented with sufficient accuracy any similar number which might be collected in any part of London, and, by fair inference, any multiple of that number to the extent of hundreds of thousands. Here, then, I possessed a true key to the "existing state of medical practice" in this country.

Having these prescriptions at my command, I submitted them to an analysis (if I may use the term) which, I am bound to say, proved to be a work of very considerable time and labor. The result arrived at after much careful noting, I will now place before you.

Whilst the pharmacopœia contains 768 medicaments simple and compound, medical men do not adopt in actual practice more than 485; and what is rather remarkable, three-fourths, or 75 per cent., of these occur less than once in every 100 prescriptions written; so that if we take the remaining fourth, or the leading remedies as they may be called, we shall find that these are prescribed three times where the rest are only prescribed once. The inference to be drawn from this is, that if a medical practitioner were to treat disease with these 120 leading medicines, according as he may select them, and no others—presuming the whole 485, now in use, to be of equal value—the "odds," if I may be allowed the expression, would be 1 to 3 against his success as compared with the practitioner who held the advantage of the entire range of remedies; but as these medicines are not all of equivalent value, as shown by the fact that 75 per cent. occur less than once in every 100 prescriptions, the advantage of the additional number above one-fourth, would be so reduced as to render the chances of the two practitioners very nearly equal. We shall see what further inference can be drawn in this direction.

It is impossible to pass over the fact that a few medicines take the lead in medical practice to the comparative exclusion and neglect of all the rest. Quinine heads the list by a long way, then Chloric Ether, Bicarbonate of Potash, Aromatic Spirit of Ammonia, Iodide of Potassium, Mercurial Pill, Compound Extract of Colocynth, and so on. Twenty-five of these medicines show an average occurrence of once in *seventeen* prescriptions, whilst those which remain, taken collectively, show an average occurrence of once in *one hundred and sixty-six* prescriptions. This is scarcely, perhaps, a fair calculation, but the difference is a very wide one, and serves to show where the greatest reliance in the power of drugs may be found to exist.

With regard to the prescriptions examined, it is well worthy of note, that of the 485 medicines ordered or prescribed, 429 are to be met with in the pharmacopœia; a result showing the desirability of a thorough knowledge and appreciation on our parts of this important work.

It is perhaps, however, in the form of simple remedies that we shall best estimate the value of the medicines prescribed by the physician. Here the number is reduced to 171, and the order of things is somewhat changed. Mercury takes the lead and stands prominently at the head of the list. Mercury, the very name of which strikes terror into the minds of nervous and timid patients, is still the foremost remedial agent employed by the medical profession.* After mercury we have potash, then bark, then opium, and then iron. If we take twenty-five of these leading simple substances, as in the case of the componnds, we shall find that 95 per cent. of all the prescriptions written contain one or more of them in some recognized form. This, I think, brings the whole matter into the smallest compass, and places us in a position to offer such further brief comments as the subject may seem to require.

Mercury, potash, bark, opium and iron. Are these medicinal substances of any service in combating the symptoms of disease? If not, the whole system of medicine is shaken, and skepticism is only too well founded. If, on the contrary, they are of service, then it is true philosophy to extend our faith, and, in the absence of certainty, or in the absence of probable injury from such a course, rely on what is possible as regards the entire scope of the Materia Medica.† Much, I think, may be said to show, in a manner sufficiently conclusive, that, with respect to disease, medicine possesses a power, the absence of which would inevitably lead to additional and prolonged suffering. But it is to be specially remembered that this power is limited. If you ask me where such limit terminates, I reply that it is beyond the scope of our present means to ascertain; but of this we may be certain, that the true value of medicine will be exactly proportionate to the skill with which the remedial power it possesses, within the limit indicated, is developed in each particular case. It is this fact

* "And yet," says Sir Thomas Watson, "we are distracted by doubts whether the powerful influence it exercises on the body be for good or for evil in the diseases for which it is given." Could anything be more eminently unsatisfactory, or more abundantly disheartening than this? Of all the medicaments in the Materia Medica, the one which is most relied on, and most frequently prescribed for the cure of sickness and disease, is still so far a puzzle and a mystery to the medical profession, that it is not known "whether the powerful influence it exercises on the body be for good or for evil in the diseases for which it is given." Well, indeed, may the natural history of disease be asked for, and an investigation into the physiological action of drugs be demanded, with a view to placing the whole therapeutical art on a surer and more scientific foundation.

† There can be no doubt that a large quantity of medicine may be taken without real injury to the system. Nature is very accommodating to substances generally regarded as highly powerful or deleterious. She rebels at first, but soon manifests a comparative indifference to their peculiar properties; as witness the atmosphere of underground railways and coal mines, containing sulphurous acid and carbonic acid gases; and the large quantities of alcohol, opium, and tobacco, consumed with apparent impunity by the people of all nations. Even arsenic may be taken in poisonous proportions, without ill effects, by the mere force of habit. The plan, therefore, of *trying* various remedies, where such is admissible, with a view to the possibility of the good to be derived from them, seems to be a wise and reasonable one in every respect.

which renders it so undesirable to follow a routine method to the exclusion, perhaps, of timely and sufficient aid. If, indeed, we look at the constancy with which certain medicines are ordered, the treatment of disease would appear, at first sight, to be *solely* a question of routine; these medicines being administered for the relief of a particular set of symptoms, because they have been found from experience to be of service in the majority of such cases. But a prescription is, or ought to be, a scientific document, the result of an adequate knowledge of the physical sciences, and a clear appreciation of all those minutiæ with regard to compound medicines which are so essential to their success. If, therefore, to write a prescription were the sole duty of the physician, the course of special study through which he passes would not be lost, but, on the contrary, would maintain all the importance which his additional duties now serve to impart. To obtain the advantage to be derived from medicine to the utmost extent of its limits, in the presence of an uncertainty which ever-varying circumstances must necessarily engender, is a work offering scope for judgment and ability of the very highest order. It is quite true that if a powerful drug, such as opium or digitalis, were given to a large number of persons, the similarity of circumstances in each instance would enable us to estimate, with a fair degree of certainty, the probable result. One stomach bears a considerable resemblance to another stomach throughout mankind. It is this similarity which renders any single remedy of known repute applicable to so many cases; and the same may be said, in a degree, of almost any drug in the Materia Medica. So far circumstances are sufficiently constant to sanction with a certain reserve, the adoption of such a mode of procedure. Universal medicines will exhibit their good effects, or supposed good effects, in a certain percentage of appropriate cases—considered appropriate as the result, solely, of experience—in which they are administered; and it is upon this principle, which is purely empirical, that drugs of probable efficacy are often—perhaps I ought to say are most frequently—recommended and taken. If, for instance, we were to select one thousand cases of ordinary cough, and administer to each the compound tincture of camphor, i. e. the paregoric of the pharmacopœia, there is every probability that 90 per cent. of such cases would be relieved thereby. True, but how many would be cured? It is not venturing too far to say that none would be cured by the medicine itself. There are few medicines, if any, whether their effects be produced by chemical, mechanical, or vital processes, that possess a direct curative action.* That which cures, call it force, vitality, or what you will, resides in the living body, and is a property, so to speak, of that body, possessed of limited power according to the relationship it bears, at any moment of time, to the laws of organized existence. The discovery to be made, therefore, is not so much the direct action of medicines in the system, if I may draw the distinction, as the exact position they individually occupy with regard to the reparative tendency which is there present. We know that medicines will produce an effect according as their properties are sedative, astringent, antacid, and so on; but we are not so well acquainted with the extent to which such effect is really useful in counteracting abnormal or faulty states of the system. Medicine will not cure, but it will often, I think, do one of two things. In either case its action is indirect. It will assist the healing power on the one hand, or it will add to it on the other. It is easy to see that this indirect action is altogether of a subordinate character. If the reparative or curative power be still in abeyance, the drug, indeed, will have done all that it was capable of doing, but the cure will be indefinitely prolonged, or it will become altogether impossible. This, I think, is where our faith has been at fault; and there would now appear to be some fear of our going to the extreme in an opposite direction. If we have expected too much from medicine, or if we have thought its virtues unbounded, the re-action may be natural, but it is scarcely philosophical. It is in the indirect, and not in the curative action of drugs that we must look for the true source of their remedial power to whatever extent they may be so imbued; and here we seem to have an approach to the secret involved in the question of the *vis medicatrix naturæ* as against the artificial treatment of disease by medicinal agents and compounds. We can assist nature and that is all. The idea is a very old one, but it has scarcely yet been fully recognized in practice. The beneficial action of quinine in ague, colchicum in gout, and arsenic in

* Dr. Bence Jones favors the chemical theory of the action of medicines in the system; while Dr. Headland thinks the vital the most plausible.

eczema, etc., is very mysterious, and quite beyond our comprehension; but it is still directed to altering a morbid condition under the guiding influence, so to speak, of constitutional agency. Hence, similar symptoms, treated with the same remedy, will disappear more rapidly in one case than in another. Where the constitution is itself the cause of disease, it will often happen that medicine will avail but little, and the symptoms will be merely palliated, but not removed. The true physician will, of course, recognize this, and act accordingly. Success in curing disease demands that diathesis and drug-action should become a united study.

Routine treatment may be available to a limited extent, for, as we have seen in the case of our paregoric, opium will relieve pain and calm irritation in almost every instance in which it is given. Other drugs will act with more or less certainty according to their respective properties. So far there is a similarty in the stomach and nervous system, and a constancy in the drug itself, which may fairly be depended upon. We know this from experience, and hence such a method of procedure is termed "empirical." But still it remains that science and positive skill will appreciate and perceive those essential points which, viewed in the light of routine merely, may be dim and uncertain. Here then is our hope. We want more light--more knowledge. If these, in the present stage of our progress, sometimes fail, as fail they will, it detracts nothing from their innate power to overcome, as far as may be, the many and great difficulties of the medical art. Let us hope that time will, in such wise, carry us farther and farther away from the region of uncertainty and doubt, and lead us to a more intimate acquaintance with the varied and ever-varying conditions of universal law.

For ourselves, let it be our constant care to see that our drugs have every chance of gaining the credit which is no doubt justly their due, and, by improved processes derived from an increasing knowledge of all that appertains to chemical, botanical, and pharmaceutical science, to assist in securing that good result to which the efforts of our physiologists are now significantly directed. So may we confidently anticipate the near approach of a time when a clearer light will appear as the reward of diligent investigation, and when "medicine will obtain the highest place among all the arts that ministers to the welfare and happiness of man."
*Chemist and Druggist.—N. Y. Journal.*

